# A Comparative Analysis of Multi‐Criteria Decision‐Making Methods and Workload Indicators of Staffing Need in Estimating the Number of Information Technology Experts Needed for District Health Networks: A Descriptive–Analytical Study

**DOI:** 10.1002/hsr2.73035

**Published:** 2026-08-16

**Authors:** Majid Jangi, Azadeh Shayan Babokani, Mohammad Hossein Masoudi, Mohsen Rezaei, Nasim Ghalili Najafabadi, Mozhgan Kazemzadeh

**Affiliations:** ^1^ Health Information Technology Research Center Isfahan University of Medical Sciences Isfahan Iran; ^2^ Department of Statistics and Information Technology Isfahan University of Medical Sciences Isfahan Iran

**Keywords:** district health network (DHN), information technology experts, multi‐criteria decision‐making (MCDM), workload indicators of staffing need (WISN)

## Abstract

**Background and Aims:**

Human resources play a vital role in healthcare service delivery and efficiency. With increasing use of information technology and growing demand for IT‐related projects, appropriate workforce planning is essential to ensure adequate staffing.

**Methods:**

This descriptive–analytical study was conducted in 2023 to estimate and compare IT workforce requirements in 27 district health networks (DHNs) affiliated with Isfahan University of Medical Sciences, Iran, using the Workload Indicators of Staffing Need (WISN) and Multi‐Criteria Decision‐Making (MCDM) approaches. A nine‐member expert group participated throughout the study in reviewing the data‐collection process, WISN activity classification and standardization, and the selection and weighting of MCDM factors.

**Results:**

WISN provided valid workforce estimates for 21 of 27 DHNs. The estimates were identical to MCDM results in 15 of 21 DHNs (71.4%). Differences between the two methods were no greater than 0.5 IT experts in 19 DHNs (90.5%), while a difference of one expert was observed in two DHNs (9.5%). Based on the estimated staffing requirements, the projected ratio would be approximately one IT expert per 145 personnel across the 27 DHNs.

**Conclusion:**

WISN and MCDM produced broadly concordant workforce estimates in most DHNs. WISN may provide a practical activity‐based approach for IT workforce planning when routine workload data are unavailable, provided that activities are systematically recorded over a representative period. MCDM can complement WISN by incorporating organizational and service‐related characteristics into workforce planning.

## 1 Introduction

Managers' primary mission in organizations is to make optimal use of human resources, facilities, and information. Among these, human resources are the most critical organizational asset because they enable the effective use of other resources [1]. Human resources in the health system also play a vital role in service delivery and effectiveness [2]. When health workers are equitably distributed and possess appropriate competencies, they are more likely to provide high‐quality care [3].

Despite the need for diverse healthcare staff, most workforce planning efforts have focused primarily on conventional clinical professions such as physicians and nurses, while other essential occupational groups have received less attention [4]. With the ongoing digital transformation of health systems, the role of information technology (IT) human resources has become increasingly prominent. Researchers emphasize that successful digital transformation depends heavily on workforce capacity rather than technology alone [5]. Given the expanding use of IT in healthcare and the growing demand for related projects, shortage of qualified IT experts has emerged as a significant managerial challenge, making accurate workforce planning a priority [6].

The Iranian health system operates across primary, secondary, and tertiary levels in both urban and rural areas. District Health Networks (DHNs), affiliated with medical universities, oversee service delivery within defined geographic regions. Each DHN supervises multiple units, including primary care facilities and comprehensive urban and rural health centers. The allocation of human resources, including Information Technology (IT) professionals, varies across DHNs according to the number and extent of units covered.

Evidence on the required number of IT professionals in healthcare systems remains limited, and most previous studies have focused specifically on Health Information Technology (HIT) personnel rather than broader IT roles [6]. For example, Hersh et al. estimated HIT workforce requirements in the United States and reported substantial future demand associated with electronic medical record expansion [7]. Subsequent analyses in the United Kingdom and Australia similarly suggested specific ratios of HIT staff to non‐IT personnel [8]. However, these findings may not be directly applicable to Iran, where the roles of IT experts and HIT personnel differ considerably in training, responsibilities, and deployment.

In Iran, HIT graduates primarily work in medical records and statistics units, whereas IT experts are responsible for information systems development, network infrastructure, and technical support. This structural distinction limits the transferability of international HIT‐based workforce estimates to the Iranian context. Although some Iranian studies have examined IT staffing using multi‐criteria decision‐making approaches or assessed administrative workforce levels [9, 10, 11, 12, 13], comprehensive estimation of IT workforce requirements in district health networks remains scarce. Insufficient planning in this area may contribute to staff overload, burnout, and reduced system efficiency [6].

Traditional workforce planning approaches based on simple ratios or descriptive indicators are often inadequate for addressing the complexity of modern health systems. To support evidence‐based staffing decisions, the World Health Organization introduced the Workload Indicators of Staffing Need (WISN) method, which estimates required staff based on actual workload and activity standards [14, 15, 16]. Although WISN has been widely applied in health workforce research, its use for estimating IT staffing faces practical challenges, particularly the limited availability of reliable activity data for IT personnel [17].

In university‐affiliated health networks, activity data for IT experts are generally not recorded. To address this, the study collected 1 month of such data and estimated staffing needs using the Workload Indicators of Staffing Need (WISN). The Multi‐Criteria Decision‐Making (MCDM) method was also applied to evaluate whether WISN results aligned with an alternative estimation approach. A focus‐group discussion identified key factors influencing IT workforce requirements for the MCDM model, supporting this comparative assessment in district health networks affiliated with Isfahan University of Medical Sciences [10].

## 2 Methods

### 2.1 Study Design and Setting

This descriptive–analytical study was conducted in 2023 to estimate and compare the number of Information Technology (IT) experts required in the 27 district health networks affiliated with Isfahan University of Medical Sciences, Iran, using two workforce‐planning approaches: the Workload Indicators of Staffing Need (WISN) method and a Multi‐Criteria Decision‐Making (MCDM) model. The primary objective was to compare the workforce requirements estimated by the two approaches when applied to the same health networks.

### 2.2 Expert Group and Methodological Consensus Process

A nine‐member expert group comprising experienced IT managers and experts from the university and affiliated health networks was established to guide and review the methodological procedures throughout the study. The members had 5–30 years of professional experience in information technology and related managerial or technical positions; the group included representatives from university management and health networks, with relevant expertise in statistics and information technology management.

Multiple meetings and consultation sessions were held throughout the study, and the procedures, methodological decisions, and intermediate findings were repeatedly reviewed and approved by the expert group. For the WISN method, the group contributed to the development and content review of the activity‐recording instrument, classification of IT activities, interpretation of activity standards, and review of workload and staffing calculations. The instrument was piloted in five health networks: an initial 2‐week pilot phase, followed by a second 1‐month pilot phase, before final implementation across all 27 networks. For the MCDM method, the group identified and reviewed potential workload determinants, assessed their relevance and discriminatory value, determined the internal and external weights, and reviewed the resulting workforce estimates. Thus, expert consultation and consensus were incorporated iteratively throughout both methods.

### 2.3 WISN‐Based Estimation of IT Workforce Requirements

The WISN method was implemented according to the World Health Organization framework. Because routine records of IT activities were not available, a structured electronic, open‐ended activity‐recording form was developed to document the type, frequency, and duration of IT activities. The form was reviewed by the expert group and initially piloted for 2 weeks in five health networks. The findings were reviewed and the form was revised; based on the activities identified in this phase, IT activities were classified into six groups. The revised form was then implemented for a second 1‐month pilot in the same five networks, further refined, and subsequently used across all 27 health networks. IT experts recorded their activities during June 2023 (1–31 Khordad 1402; May 22–June 21, 2023).

Recorded activities were classified into six groups: software, hardware, information security, server support, network support, and meetings/coordination. These activities were mapped to the three WISN workload categories: main, support, and additional activities. Because the frequency and duration of all three categories were directly recorded during the study period, support and additional activities were quantified directly rather than estimated solely through conventional allowance factors (similar to main activities).

Available working time (AWT) was calculated by accounting for working days, public holidays, authorized leave, sick leave, and other permitted absences [15]. A further 10% of working time was deducted for personal needs and fatigue allowance. The resulting AWT during the study month was 125.1 h.

For each activity, the activity standard was defined as the time required by a trained and motivated worker to perform the activity according to accepted professional practice. To reduce the influence of outliers, recorded activity times were clustered using the K‐means method [18], and the mean of the appropriate cluster was used as the activity standard.

In the WISN framework, the standard workload is the amount of work that a worker can perform during the available working time based on the activity standard. The WISN manual explicitly notes that this calculation assumes a health worker devotes their entire annual working time to a single workload component, an assumption the manual itself acknowledges is not true in real life [15]. In this study, the two‐step calculation this assumption underlies (deriving a standard workload, then dividing actual workload by it) was therefore algebraically reformulated into a single, direct step that does not require explicitly invoking this idealized full‐time assumption, and the reformulation was proven to be mathematically equivalent to the original WISN calculation:

(1) $$\text{Number of forces required}\;=\frac{\text{actual workload}}{\text{standard workload}}=\frac{\text{actual workload}}{\frac{\text{available time}}{\text{activity standard}}}=\;\frac{\mathrm{actual}\;\mathrm{workload}\;*\;\text{activity standard}\;}{\text{available time}}\;$$

This formulation is mathematically equivalent to dividing actual workload by standard workload, because standard workload is calculated as available working time divided by activity standard. The numerator represents the total time required to perform the observed workload, and the denominator represents one worker's available working time; dividing one by the other therefore directly gives the number of workers required for that activity. This reformulation provides a more direct route from the recorded workload to this staffing requirement, without separately computing or interpreting the idealized standard‐workload quantity described above, and was used as a methodological clarification of the standard‐workload calculation, without modifying the underlying WISN framework.

The actual workload of each employee (the frequency of each activity) was standardized to 25 available working days when the number of days with recorded activities differed from the full study period. For each IT expert, the activity‐specific workforce requirements (calculated using Formula 2) were then summed to obtain the initial staffing requirement [15].

Allowance factors were subsequently incorporated. A group allowance factor of 20% was applied to account for urgent and common activities, including implementation of new hardware and software systems. An individual allowance factor was calculated for each health network based on the proportion of available working time spent traveling to its covered units. The final workforce requirement for each health network was obtained by combining the activity‐based staffing requirements with the relevant individual and group allowances [15].

The workload pressure ratio was calculated as the number of available IT experts divided by the estimated number required. A ratio below 1 indicated a relative staffing shortage and higher workload pressure, a ratio of 1 indicated approximate balance, and a ratio above 1 indicated a relative staffing surplus [15].

### 2.4 MCDM‐Based Estimation of IT Workforce Requirements

The MCDM model was developed as an independent approach [19] based on organizational infrastructure and service scope to estimate IT workforce requirements. Potential determinants of IT workload were identified and reviewed through the expert‐group discussions. Initially, five factors were considered: the number of covered units, number of computers, average distance of covered units from the network headquarters, number of staff users of IT systems, and number of health information software applications. Through structured discussion and expert consensus, the number of staff users was excluded because of its substantial overlap with the number of computers, while software applications were excluded because similar applications were implemented across the health networks and therefore had limited discriminatory value. The three retained factors were the number of covered units, number of computers, and average distance of covered units from the network headquarters.

Each retained factor was divided into empirically defined classes reflecting increasing workload, and an internal weight (*W_i_*) was assigned to each class [19]. The relative importance of the three factors was then determined through expert consensus using external weights (*α_i_*) of 5.5 for the number of computers, 2.0 for the number of covered units, and 2.5 for the average distance from the network headquarters.

The Simple Additive Weighting (SAW) approach was used to combine the internal and external weights and generate a final score for each health network [19]. The final score represented the relative priority of the network in terms of IT workforce need, with higher scores indicating greater relative staffing need.

Because the MCDM score represented relative rather than absolute staffing need, two health networks considered by the expert group to have an appropriate staffing level according to the WISN results were identified as reference networks. The relationship between their final scores and existing IT staffing was used for calibration. The resulting calibration coefficient was 2.004, and each network's final score was divided by this coefficient to estimate its required number of IT experts.

### 2.5 Comparison of the Two Methods

The primary comparison was performed at the health‐network level. For networks with valid estimates from both methods, the estimated number of required IT experts was compared directly. The analysis examined the magnitude and direction of differences between WISN‐ and MCDM‐based estimates and the proportion of networks for which the two approaches produced identical or different estimates.

Estimated workforce requirements were also compared with the number of currently available IT experts to identify relative staffing shortages, approximate balance, or surpluses. For the WISN estimates, the workload pressure ratio was used to characterize the relationship between available and required IT personnel. In descriptive results, continuous variables were summarized using means and standard deviations when appropriate, and categorical variables using frequencies and percentages. Data processing and descriptive calculations were performed using Microsoft Excel 2016, while K‐means clustering was performed using IBM SPSS Statistics version 25.

## 3 Results

### 3.1 Expert Group

Nine IT managers and experts participated in the focus‐group sessions. Three (33%) were women and six (67%) were men. Their professional experience ranged from 5 to 30 years (mean 15.1 ± 7.6 years). Four (44%) worked in university management and five (56%) were responsible for IT units in health networks (Table 1).

**Table 1:** Frequency and percentage of demographic variables of Delphi members (focus group).

Variable	Group	Frequency	Percentage
Sex	Man	6	67%
	Woman	3	33%
Educational level	Bachelor's degree	4	44%
	Master's degree	5	56%
Workplace	University management area	4	44%
	Health networks	5	56%
Educational qualifications	Computer‐software	6	67%
	Computer‐hardware	1	11%
	Computer‐information technology	1	11%
	Computer‐machine intelligence robotics	1	11%

### 3.2 Results of the WISN Method

The available time of IT experts after subtracting permitted absences in May–June 2023 was calculated to be 125.1 h (Table 2). The six major groups identified for the activities of IT experts of health networks were summarized as software activities (45% of the workload), hardware (14%), security (1%), server support (1%), network support (11%), and meetings and coordination (28%) (Table 3). Among the 27 health networks, 21 had sufficient activity records for WISN‐based estimation. Of these, 13/21 (61.9%) had an estimated shortage of IT experts, with the shortage ranging from 0.5 to 1 expert and workload‐pressure ratios of 0.67–0.83. Four of 21 (19.0%) networks had approximately sufficient staffing (ratio = 1.0), while 4/21 (19.0%) had a surplus of 0.5 experts (ratio = 1.33) (Table 4).

**Table 2:** IT experts' available time in Khordad*.

Time variables	Time
Total number of days in Khordad	31 days
Weekends and holidays	6 days
Available working days	25 days
Available working hours	158.5 h
Earned leave	2.5 days
Sick leave (average number of sick days for IT network personnel in Khordad)	0.2 days
Permitted unemployment (10%) (40 min per day)	8 h
Available working hours after subtracting above permitted absences	125.1 h

* Data collection was conducted from the 1st to the 31st of Khordad 1402 in the Iranian calendar, which corresponds to May 22–June 21, 2023, in the Gregorian calendar. This alignment ensures accurate representation of the study period for international readers.

**Table 3:** Components of IT expert's workload in health networks.

Field of activity	Type of activity	Number of sub‐activities	Frequency of recorded activity	Percentage of recorded time	Recorded time (hours)	Percentage of recorded activity frequency
Deployment/implementation/debug/tracking problems (updating all software and systems)	Main	14	1606	43%	1664	45%
Checking the security of existing systems and servers according to the instructions issued by the university	Main	3	31	1%	51	1%
Installation and commissioning, as well as technical support of hardware equipment (service/repair/maintenance/technical inspection/parts replacement)	Main	9	511	16%	610	14%
Support for existing servers	Support	2	18	1%	32	1%
Support and maintenance of network communications (intranet, wireless)	Support	6	390	14%	533	11%
Other (meetings/training/writing letters/preparing and sending reports/coordination/responsibility/cooperation with other units)	Additional	5	978	24%	937	28%
Total		39	3534	100%	3827	100%

**Table 4:** Final score and number of IT staff required for 27 health networks under the coverage of Isfahan University of Medical Sciences based on the explained model.

MCDM							WISN									
Health networks	Number of IT experts	Final score	Forecast based on standard	Approximate final number of forces	The current and predicted difference	Working pressure	Actual workload* Activity standard	Standard workload	Initial estimate	Group allowance*	Individual allowance	Final estimate	Approximate final number of experts	The difference between WISN and the current number	Working pressure	Difference in estimates between the two methods
No. 4	1	2.42	1.21	1.5	−0.5	0.67	127.83	125.1	1.02	0.21	0.2	1.43	1.5	−0.5	0.67	0
No. 5	1	2.47	1.23	1.5	−0.5	0.67	121.01	125.1	0.97	0.21	0.08	1.26	1.5	−0.5	0.67	0
No. 6	1	2.47	1.23	1.5	−0.5	0.67	139.5	125.1	1.12	0.21	0.18	1.5	1.5	−0.5	0.67	0
No. 7	1	2.53	1.26	1.5	−0.5	0.67	157.97	125.1	1.26	0.21	0.2	1.67	1.5	−0.5	0.67	0
No. 11	1	2.85	1.42	1.5	−0.5	0.67	145.05	125.1	1.16	0.21	0.02	1.39	1.5	−0.5	0.67	0
No. 13	1	2.86	1.43	1.5	−0.5	0.67	126.17	125.1	1.01	0.21	0.04	1.26	1.5	−0.5	0.67	0
No. 14	1	2.86	1.43	1.5	−0.5	0.67	134.5	125.1	1.08	0.21	0.16	1.44	1.5	−0.5	0.67	0
No. 23	2	4.87	2.43	2.5	−0.5	0.8	135.01	125.1	1.08	0.21	0.02	2.62	2.5	−0.5	0.8	0
No. 24	2	5.03	2.51	2.5	−0.5	0.8	98.85	125.1	0.79	0.21	0.165	2.33	2.5	−0.5	0.8	0
No. 2	1	2.18	1.09	1	0	1	114.24	125.1	0.91	0.21	0.02	1.14	1	0	1	0
No. 22	2	4.06	2.02	2	0	1	101.17	125.1	0.81	0.21	0.079	2.2	2	0	1	0
No. 15	2	2.89	1.44	1.5	+0.5	1.33	137.39	125.1	1.1	0.21	0.12	1.43	1.5	+0.5	1.33	0
No. 17	2	3.08	1.54	1.5	+0.5	1.33	122.57	125.1	0.98	0.21	0.18	1.37	1.5	+0.5	1.33	0
No. 18	2	3.12	1.56	1.5	+0.5	1.33	117.31	125.1	0.94	0.21	0.14	1.29	1.5	+0.5	1.33	0
No. 16	2	2.95	1.47	1.5	+0.5	1.33	149.70	125.1	1.20	0.21	0.06	1.47	1.5	+0.5	1.33	0
No. 3	1	2.18	1.09	1	0	1	124.81	125.1	1	0.21	0.09	1.3	1.5	−0.5	0.67	0.5
No. 9	1	2.62	1.31	1.5	−0.5	0.67	102.55	125.1	0.82	0.21	0.12	1.15	1	0	1	0.5
No. 19	1	3.29	1.64	2	−1	0.5	165.04	125.1	1.32	0.21	0.05	1.58	1.5	−0.5	0.67	0.5
No. 21	2	3.95	1.97	2	0	1	109.03	125.10	0.87	0.21	0.05	2.26	2.5	−0.5	0.8	0.5
No. 26	4	9.27	4.62	5	−1	0.8	99.6	125.1	0.80	0.21	0.02	4.12	4	0	1	1
No. 27	5	9.38	4.68	5	0	1	116.25	125.1	0.93	0.21	0.10	6.2	6	−1	0.83	1
No. 1	1	2.14	1.07	1	0	1	—	125.1	—	0.21	—	—	—	—	—	—
No. 8	1	2.61	1.3	1.5	−0.5	0.67	—	125.1	—	0.21	—	—	—	—	—	—
No. 10	1	2.63	1.31	1.5	−0.5	0.67	—	125.1	—	0.21	—	—	—	—	—	—
No. 12	1	2.85	1.42	1.5	−0.5	0.67	—	125.1	—	0.21	—	—	—	—	—	—
No. 20	1	3.64	1.81	2	−1	0.5	—	125.1	—	0.21	—	—	—	—	—	—
No. 25	3	5.16	2.58	2.5	+0.5	1.2	—	—	—	0.21	—		—	125.1		—

### 3.3 Results of the MCDM Method

The factors and weights used to develop the MCDM model, including the internal (*W_i_*) and external (*α_i_*) weights determined by the expert group, are reported in Table 5. After calibration of the MCDM scores using the reference networks, 16/27 (59.3%) health networks were classified as having a shortage of IT staff, with estimated shortages ranging from 0.5 to 1 expert and workload ratios of 0.5–0.8. Six of 27 (22.2%) networks had sufficient staffing, while 5/27 (18.5%) had a surplus of IT staff (Table 4). Currently, 16/27 (59%) networks had one IT staff member, 8/27 (30%) had two, and 3/27 (11%) had more than two IT staff members. Across the 27 networks, 44 IT experts served 7415 personnel, corresponding to a ratio of 1:168; based on the estimated staffing requirements, this ratio would improve to approximately 1:145.

**Table 5:** Classification and internal weighting of influential factors in determining the number of IT staff in health networks.

Factor	Groups	No. of health networks	Weight (*W*_*i*_)	Factor	Groups	No. of health networks	Weight (*W*_*i*_)
Number of computers (*α*_1_ = 5.5)	0–99	4	0.04	The number of networks covered (*α*_1_ = 2.5)	20–29	6	0.12
	100–174	6	0.06		30–39	6	0.16
	175–249	6	0.09		40–49	5	0.20
	250–324	4	0.14		50–59	4	0.25
	325–399	1	0.19		60–69	1	0.31
	400–499	2	0.27		70–79	2	0.38
	500–599	2	0.36		80–89	0	0.45
	600–699	0	0.48		90–99	0	0.54
	700–799	0	0.62		100–109	1	0.62
	800–899	1	0.80		110–119	0	0.71
	900–1000	1	1.00		120–129	0	0.79
The average distance from headquarters to networks (*α*_2_ = 2)	1–10 km	3	0.62		130–139	0	0.87
	10–20 km	9	0.68		140–149	1	0.94
	20–30 km	5	0.75		150–160	1	1.00
	30–40 km	5	0.83				
	40–50 km	4	0.91				
	50 km and above	1	1.00				

### 3.4 Comparison of WISN and MCDM Results

Among the 21 health networks with valid WISN estimates, the two methods produced identical workforce estimates in 15/21 networks (71.4%). The estimates differed by 0.5 expert in 4/21 networks (19.0%) and by one expert in 2/21 networks (9.5%). Thus, the difference between the two methods was no greater than 0.5 expert in 19/21 networks (90.5%) (Table 4).

## 4 Discussion

This study compared IT workforce requirements estimated by WISN and MCDM in district health networks. The two methods produced concordant estimates for the large majority of networks, with only small differences in the remainder, indicating that despite relying on different types of input data, WISN and MCDM captured broadly comparable IT workforce requirements.

The two methods approach workforce estimation from different perspectives. WISN is directly workload‐based and incorporates the frequency and duration of actual activities, whereas the MCDM model developed in this study uses selected organizational and infrastructure‐related characteristics. WISN therefore provides a direct representation of observed workload but requires detailed and reliable activity data. MCDM can be useful when such data are unavailable, although it may not capture all variations in actual work patterns. The similarity observed between the two approaches suggests that measurable infrastructure and service‐scope characteristics may provide useful information for workforce planning.

In the MCDM method, the factors affecting the workload of IT personnel in healthcare networks were first identified and weighted in focus group sessions by experts in the field. The number of computers received the highest external weight (5.5), followed by the average distance of covered units from the network headquarters (2.5) and the number of covered units (2.0). These findings highlight the importance of IT infrastructure and geographic service scope in determining workforce requirements. In 2017, Antonio Miguel Cruz and Mayra R. Guarin also showed, by fitting a multivariate regression model, that the total number of devices and the total hours allocated to these devices directly impact the number of clinical engineers in hospital wards [20]. In the study by Jangi et al., which assessed the IT capacity of Isfahan hospitals, the number of active computers and their peripherals also received a high external weight [10].

The geographic scope of service provision is particularly relevant to district health networks, where dispersed facilities may increase the time required for IT support. In the study by Jangi et al., the “distance to the furthest unit receiving IT services” was among the practical factors identified in determining the number of IT experts required by hospitals [10]. In a systematic review done by Pirotta et al. in 2022, while the absence of a gold standard for determining the required number of health personnel is demonstrated, they emphasize that to address the personnel needs in the health system, it seems necessary to develop a tool or model that considers a sufficient number of variables to identify the characteristics of the overall context [21].

This study represents, to our knowledge, the first application of the WISN method specifically for planning the recording, categorization, and timing of IT activities in health networks. The prerequisite for implementing this method is annual recorded statistics, including activities and their time. Given that, this method is considered relatively simple compared to previous methods and has been widely used [15]. In university‐affiliated units, including health networks, statistics on the activities of IT experts are not recorded or documented either formally or in this study, the absence of routine activity records was addressed by developing and pilot‐testing an activity‐recording process before implementing WISN across the participating networks. The repeated review of procedures and findings by the nine‐member expert group throughout the study further supported the methodological consistency and practical relevance of the process.

Several limitations should be considered. WISN estimates could be calculated for only 21 of the 27 networks because sufficient activity records were unavailable for the remaining networks. The workload data also covered 1 month rather than a full year, which may limit their representativeness of annual workload patterns. In addition, awareness of the purpose of recording may have influenced reporting behavior. In some networks, simultaneous service provision or incomplete, implausible, or excessive recording may have affected the estimates. These limitations highlight the importance of data quality when applying WISN to IT activities for which routine workload statistics are unavailable.

Despite these limitations, the similarity between the two methods is noteworthy. Exact estimates were identical in 15/21 networks (71.4%), while differences were no more than 0.5 expert in 19/21 networks (90.5%). This similarity should not be interpreted as evidence that either method is more accurate, because no independent reference standard was available for determining the true staffing requirement. Rather, the findings suggest that the selected organizational and infrastructure characteristics used in MCDM may capture important dimensions of IT workload that are also reflected in activity‐based WISN estimates. These findings further suggest that when the variety of activities performed over a short recording period resembles that observed over a full year, planning for short‐term, well‐structured activity recording may be a feasible alternative to a full annual audit, and that WISN estimates can remain informative even when staff are aware that their activities are being recorded for a staffing assessment.

The practical implications are important for workforce planning. Where reliable activity data are available, WISN can provide a direct workload‐based assessment of staffing requirements. Where such data are unavailable or costly to collect, MCDM may provide a practical initial estimate based on readily measurable organizational characteristics. Using the two approaches together may also provide a useful cross‐check for workforce planning decisions, particularly in geographically dispersed health networks.

Another strength of this study is that Formula 2 removes ambiguity from the step of “calculating the standard workload for each of the main activities” by expressing it directly in measurable terms. The algebraic reformulation presented in Formula 2 provides a more transparent expression of the standard‐workload calculation when the activity standard is expressed as time per activity. By linking actual workload, activity time, and available working time directly, the formulation clarifies how the observed workload translates into the proportion of one worker required. This reformulation does not modify the underlying WISN framework but may facilitate its interpretation and application to IT activities and other occupations in which workload is recorded as activity frequency and activity standards are expressed in units of time.

## 5 Conclusion

In this study, WISN and MCDM produced broadly concordant estimates of IT workforce requirements across the healthcare networks. Identical estimates were obtained in 15 of 21 networks (71.4%), while differences were no more than 0.5 IT expert in 19 of 21 networks (90.5%), indicating a relatively high level of consistency between the two approaches. WISN provides a direct workload‐based estimate when reliable activity data can be collected, whereas MCDM can provide a practical estimate based on measurable organizational and infrastructure characteristics when detailed workload data are unavailable.

The findings also suggest that WISN can be practically implemented when historical activity records are unavailable by using a structured activity‐recording process with expert review and validation. The algebraic reformulation of the standard‐workload calculation further clarifies the application of WISN to activities expressed in units of time. Therefore, the choice between WISN and MCDM should be guided by the availability and quality of workload data, the characteristics of the workforce, and the resources available for data collection and expert assessment. Using the two approaches together may provide a complementary basis for workforce planning and support more informed staffing decisions in geographically dispersed healthcare networks.

## Author Contributions

**Majid Jangi:** funding acquisition, methodology, conceptualization, project administration, supervision, validation. **Azadeh Shayan Babokani:** supervision, software. **Mohammad Hossein Masoudi:** supervision, software, validation. **Mohsen Rezaei:** formal analysis, validation. **Nasim Ghalili Najafabadi:** software, data curation. **Mozhgan Kazemzadeh:** validation, visualization, writing – original draft, writing – review and editing, software, formal analysis, data curation, resources, methodology, supervision, investigation.

## Funding

The authors have nothing to report.

## Ethics Statement

This study was ethically approved by the Ethical Committee of Isfahan University of Medical Sciences with the ethical code of IR.MUI.NUREMA.REC.1401.153. No individual patient data was collected in the course of this study. IT expert participation was voluntary. Information on study purpose, process, and outputs was provided to the panelists through a webinar. Verbal Informed consent was obtained from all panelists before their participation in this Delphi panel.

## Conflicts of Interest

The authors declare no conflicts of interest.

## Transparency Statement

The lead author Mozhgan Kazemzadeh affirms that this manuscript is an honest, accurate, and transparent account of the study being reported; that no important aspects of the study have been omitted; and that any discrepancies from the study as planned (and, if relevant, registered) have been explained. All authors have read and approved the final version of the manuscript. Mozhgan Kazemzadeh, the corresponding author, had full access to all of the data in this study and takes complete responsibility for the integrity of the data and the accuracy of the data analysis.

## Data Availability

The data that support the findings of this study are available from the corresponding author upon reasonable request.
